# Association Between Heavy Metal Exposure and Autism Spectrum Disorders: Discrepancies, Research Gaps, and Future Priorities of Human Epidemiological Studies

**DOI:** 10.1029/2026GH001955

**Published:** 2026-07-31

**Authors:** Aporajita Das Trisha, Md. Ashik Imran, Jaasia Momtahena Hafsa, Nayan Chandra Mohanto

**Affiliations:** ^1^ Department of Biochemistry and Molecular Biology Shahjalal University of Science and Technology Sylhet Bangladesh

**Keywords:** heavy metals, arsenic, cadmium, lead, mercury, autism spectrum disorder

## Abstract

Autism spectrum disorder (ASD) in children is a major public health challenge. The potential epidemiological links between heavy metal exposure and ASD remains a controversial issue. This critical review examines the epidemiological discrepancies, including variations in exposure assessment, study design, and population differences that contribute to the ongoing debate. Key research gaps, such as the need for longitudinal studies and mechanistic insights, are addressed. Finally, we outline future priorities to advance understanding of heavy metals' role in ASD. Lead (Pb), cadmium (Cd), mercury (Hg), and arsenic (As) were selected heavy metals. The PubMed and Scopus databases, as well as the Google Scholar search engine, were searched to retrieve original research articles on human epidemiological studies that utilized the selected “exposure keywords” in conjunction with the “outcome keywords.” Finally, 36 full‐length articles, irrespective of age, sex, regions, and race/ethnicity, were included for the present review. We revealed inconsistent associations between prenatal and childhood urinary, blood, and hair, As and Cd exposure, and ASD outcomes. In contrast, elevated prenatal and early childhood Pb and Hg concentrations in blood and hair samples showed a significant consistent association with both increased ASD risk and symptom severity, even after adjustment for key demographic and environmental confounders. The findings are inconsistent across metals and studies, and should be interpreted with caution due to potential residual confounding and heterogeneity in exposure assessment methods. Large prospective cohort studies are needed to clarify causal relationships. The path analysis of relevant biomarkers is also warranted to establish biological mechanism.

## Plain Language Summary

This review examined whether exposure to lead, cadmium, mercury, and arsenic is linked to autism spectrum disorder (ASD) by analyzing 36 human studies. The findings were metal‐specific. For arsenic and cadmium, most studies found no clear association. Mercury results were highly variable: about half reported positive links, while others found none or inverse relationships. Lead showed the most consistent signal, with 60% of studies linking higher levels to increased ASD risk or severity, although many well‐conducted studies found no association. Overall, the evidence does not support a blanket conclusion that heavy metals cause ASD. Rather, lead and mercury may contribute to ASD risk in certain populations or under specific exposure conditions, but these findings require cautious interpretation due to methodological differences and potential biases. Long‐term prospective mother‐child cohort studies with significant participants are needed to clarify whether these metals truly play a causal role.

## Key Points

- Pb and Hg showed positive ASD links in 55%–60% of studies; As and Cd mostly inconsistent
- No studies assess joint metal mixture effects using advanced multipollutant models
- Need large birth cohorts with repeated biomonitoring across prenatal to early childhood

## 1 Introduction

Autism spectrum disorders (ASDs) are characterized by impairments in social interaction and communication along with persistent repetitive behaviors, restricted activities, sensory hypersensitivity, language delays, and intellectual disabilities (Barbaresi et al., 2005; Fiore et al., 2020; Guze, 1995). It is the most detrimental form of neurodevelopmental disorder affecting people of all age groups worldwide (Morris‐Rosendahl & Crocq, 2020). The prevalence and incidence rate of ASD was increased worldwide over the years especially in USA where the rate was drastically increased in 2014 (1/68) than that of 1980–1990 (4–5/10,000); whereas the prevalence of 8 years UK children was 1/64 (Baio, 2018; Barbaresi et al., 2005; Metwally et al., 2015). Globally, around 52 million ASD cases were reported, with approximately five times higher severity in boys than in girls (Baxter et al., 2015; CDC, 2021). It is believed that sociodemographic and lifestyle factors, nutritional alterations, immunological influences, genetic and epigenetic changes, and exposure to persistent and non‐persistent environmental xenobiotics might play a pivotal role in the development of ASD (Deisher et al., 2015; Fido & Al‐Saad, 2005; Jumah et al., 2016; Khaled et al., 2016; Lakshmi Priya & Geetha, 2011; Mohamed et al., 2015; Nabgha‐e‐Amen et al., 2020; Yassa, 2014). Among the large group of environmental chemicals, heavy metals are persistent in the environment with high longevity, and largely bioaccumulate once they enter the human body (Briffa et al., 2020; Heng et al., 2022; Nabgha‐e‐Amen et al., 2020). Humans are exposed to these heavy metals through the consumption of food items (Khan et al., 2022), marine animals (Han et al., 2021), drinking contaminated water (Islam et al., 2015), and inhalation of polluted air (Al‐Harbi et al., 2021). Human urine, blood, nail, and hair matrices are generally used for human biomonitoring of heavy metal exposure levels (Adams et al., 2013; Adams et al., 2017; Alampi et al., 2021; Fiore et al., 2020; Geier et al., 2012; Gil‐Hernández et al., 2020; Lakshmi Priya & Geetha, 2011; Nabgha‐e‐Amen et al., 2020). Heavy metals may cause neurodevelopmental and behavioral abnormalities, acting as neurotoxins (Harchaoui et al., 2020; Nabgha‐e‐Amen et al., 2020).

A significant number of human epidemiological studies relating to heavy metals biomonitoring, and their associations with ASD have been conducted, taking into account the increasing number of ASD cases, and the ultimate public health burden (Abd Wahil et al., 2022; Blaurock‐Busch et al., 2012; Fiore et al., 2020; Golding et al., 2018; Hodgson et al., 2014; Lakshmi Priya & Geetha, 2011; Yau et al., 2014). To resolve the question of whether the evidence of associations between heavy metal exposure and the development of ASD is consistent, a precise and brief overview of the overall epidemiological findings is required. It cannot be denied that the role of environmental factors in ASD has already been explored in some previously published review articles. However, most of these considered a wide range of environmental toxicants without strictly focusing on heavy metals, and/or only a single metal, and/or limited assessment period or age, and/or focused on the causes of ASD, and/or on the mechanism (Balali‐Mood et al., 2021; Bjørklund et al., 2018; Dickerson et al., 2017; Fujiwara et al., 2016; Gorini et al., 2014; Kalkbrenner et al., 2014; Ratajczak, 2011). This review was conducted to summarize and explore all types of associations between single and combined exposure to heavy metals and ASD, its severity, and risk at all age stages of life. This critical review examines the epidemiological discrepancies, including variations in exposure assessment, study design, and population differences that contribute to the ongoing debate. Key research gaps, such as the need for longitudinal studies and mechanistic insights, are identified. Finally, we outline future priorities to advance understanding of heavy metals' role in ASD.

### 1.1 Mechanistic Overview

Though a few plausible mechanisms were suggested to explain how heavy metals may induce neurodevelopmental disorders, the explicit role in the development and pathogenesis of ASD is still unclear. It has been reported that chronic exposure to heavy metals might inhibit the antioxidant activities by blocking the sulfhydryl group of antioxidant molecules, increase oxidative stress, and reduce the detoxifying activity of different tissues, including the brain (Al‐Ameen et al., 2020; Tarro et al., 2019). They also induce mitochondrial dysfunction, neuroinflammation, and epigenetic dysregulation, which are increasingly recognized as key biological pathways implicated in ASD pathophysiology (Hill et al., 2015; Siddiqui et al., 2016). This sequence of events might influence the neurobehaviors during children's early development and lead to an increased risk of ASD (Zaky, 2017).

Toxicological studies evident that heavy metals especially Pb and Hg can easily cross the blood‐brain barrier during gestation and early childhood, thereby interfering with critical developmental processes in the fetal and infant brain (Abdullah et al., 2012; Alabdali et al., 2014; Grandjean & Landrigan, 2006). The developing brain is particularly vulnerable to toxic insults during prenatal and early postnatal periods because of its high metabolic demand, immature antioxidant defense systems, and ongoing neuronal proliferation, migration, synaptogenesis, and myelination (Grandjean & Landrigan, 2014; Hill et al., 2015; Rodier, 1995).

**Figure 1:**
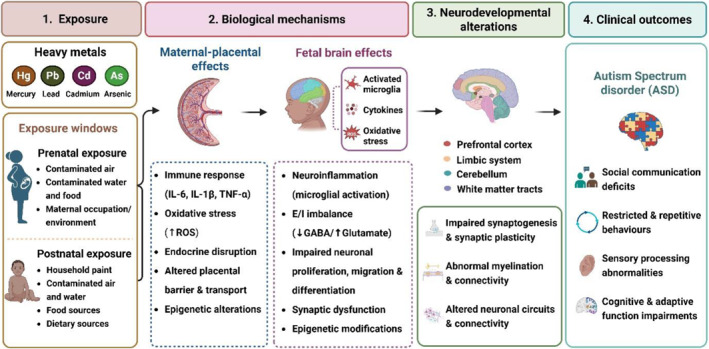
The mechanistic overview of prenatal and postnatal heavy metal exposure in the development of ASD in children through biological pathways. Figure generated by Biorender.

The schematic illustration (Figure 1) shows how prenatal or postnatal exposure to heavy metals might contribute to ASD development through multiple interconnected pathways. Prenatal exposure evident to trigger maternal immune activation, characterized by elevated levels of pro‐inflammatory cytokines (IL‐6, IL‐1*β*, and TNF‐*α*), placental immune response, and altered fetal brain cytokines (Błażewicz & Grabrucker, 2022). Heavy metals may additionally disrupt placental function by inducing oxidative stress, inflammatory signaling, endocrine dysregulation, and impaired placental transport and barrier integrity, potentially altering fetal exposure to inflammatory mediators and toxicants (Wang et al., 2009; Zhou et al., 2025).

Postnatal exposure to heavy metals may promote excessive production of reactive oxygen species and reduce antioxidant defense capabilities, leading to oxidative stress (Bjørklund et al., 2020; J. Zhang et al., 2021). It may subsequently damage DNA, proteins, and lipids, disrupt mitochondrial bioenergetics, and impair normal cellular signaling pathways (Bjørklund et al., 2020). These processes may further contribute to microglial activation, chronic neuroinflammation, excitatory/inhibitory neurotransmitter imbalance, synaptic dysfunction, and altered neuronal connectivity, all of which have been implicated in ASD‐associated neurodevelopmental abnormalities (Chauhan & Chauhan, 2015; Estes & McAllister, 2015). Such alterations may ultimately contribute to abnormal neurodevelopment and brain function including ASD (J. Zhang et al., 2021). Taken together, these findings support a multifactorial model in which heavy metal exposure may interact with genetic susceptibility and other environmental risk factors to disrupt neurodevelopmental processes across the maternal–placental–fetal axis and developing brain, contributing to ASD.

## 2 Materials and Methods

### 2.1 Selections of Heavy Metals

In most cases, density is considered the defining factor for heavy metals since there is no clear definition of what a heavy metal is. They are generally defined as those having a specific density of more than 5 g/cm^3^ (Azeh Engwa et al., 2019; Järup, 2003; Yadav et al., 2019). The World Health Organization (WHO) reported 10 chemicals as major public health concerns, among them four are heavy metals, namely As, Pb, Cd, and Hg (WHO, 2023). Environmental researchers also agreed that heavy metal exposure‐related main threats to human health are enforced by As, Cd, Pb, and Hg owing to their high degree of toxicity (Tchounwou et al., 2012). These four metals have been extensively studied, and their adverse effects on human health regularly reviewed by international bodies like the Centers for Disease Control and Prevention (CDC) and the WHO (Järup, 2003). Therefore, As, Cd, Pb, and Hg were selected as the potent heavy metals to conduct this review.

### 2.2 Literature Searching, Identification, and Screening

**Figure 2:**
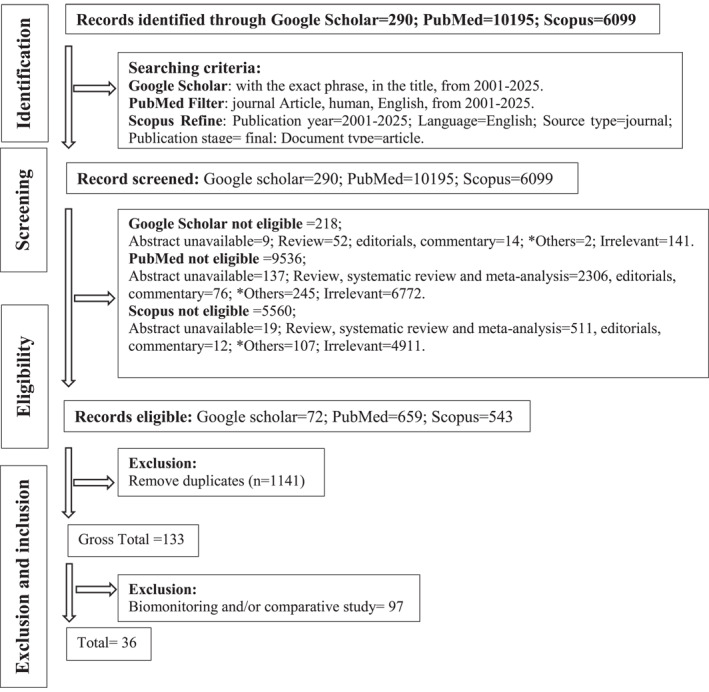
Schematic diagram of study selection. *Other includes clinical trial, controlled trial, animal studies, and cell line studies.

Google Scholar search engine, and PubMed and Scopus database were searched to find original full length and short communications research articles of human epidemiological studies using the exposure keywords namely “heavy metals,” “toxic metals,” “arsenic,” “cadmium,” “mercury,” and “lead” in all combinations with the outcome keywords including “Autism,” “Autistic spectrum disorder,” “Autism spectrum disorder,” “Asperger syndrome,” “Pervasive Development Disorder‐Not Otherwise Specified,” “Rett syndrome,” and “Childhood disintegrative disorder.” Additionally, PubMed filtering for journal articles, the inclusion of humans and English, and Scopus refining were performed to identify pertinent articles. Additionally, the reference lists of retrieved articles were also searched to find an appropriate article. Finally, the total number of records identified through Google Scholar = 290; PubMed = 10,195; and Scopus = 6,099 (Figure 2). Two authors (MAI and JMH) independently searched the respective databases and retrieved the records. The first author (ADT) was cross‐checked, screened the eligibility, and validated the records.

### 2.3 Inclusion and Exclusion Criteria

All full‐length and short communications of original research articles from all over the world, irrespective of age, sex, geographic regions, and race/ethnicity, were included in this review (Figure 2). The inclusion criteria were (a) epidemiological studies (cohort, cross‐sectional, and case‐control); (b) all life‐stage exposure to selected heavy metals and ASDs assessment (both assessment tools or clinical diagnosis); (c) human biomonitoring of selected heavy metals in biospecimens (urine, blood, hair and nails) not in environmental components; (d) published from the year 2001 to 2025; (e) consider all types of statistical associations (positive, negative and null); (f) evaluate either single or mixture exposure effect, or both; and (g) must be written in English. All other articles were excluded from this study (Figure 2).

We excluded some of the retrieved articles for the following reasons: abstract not available; involved clinical trials; controlled trials; review study, systematic review, and meta‐analysis; cell line studies; animal studies; editorial, commentary, protocol and approach, hypothesis, and letter. The articles that did not investigate the association of heavy metals with ASDs only compare the exposure levels between ASD and non‐ASD participants or investigated the associations with other disorders, and simple human biomonitoring and ecological studies were considered irrelevant and then also excluded (Figure 2).

Finally, a total of 36 articles were selected and summarized in this review based on study design. Two authors (MAI and ADT) independently prepared the summary presented in Table 1. The summary includes the information on study design, participants, ASD assessment or diagnosis, heavy metal exposure levels in each study, covariates, and key findings. The first author (ADT) cross‐checked the inclusion and exclusion criteria, followed by the key findings as shown in Table 1. All directions of associations (positive, negative, and null) for single and/or mixture metal exposure with ASDs were considered to avoid selection bias.

**Table 1:** Summary of the Human Epidemiological Studies That Investigated the Associations Between Heavy Metals and ASD

Ref.	Study design (country), subjects, age (n)	Metals and specimens (assessment tools)	Exposure levels (mean/median)	ASD diagnosis or identification	Outcome (age)	Covariates	Key findings
Cohort studies (*N* = 6)							
Alampi et al. (2021)	Cohort (Canada) Mother‐child, Mother age: 18–≥40 years, Gestation weeks: 6–13, (478)	As, Cd, Hg, and Pb Whole blood (GC‐MS)	GM (μg/L): As: 0.82, Cd: 0.20, Pb: 6.33, Hg: 0.62	SRS‐2	3‐4 years	1a, 2a, 3a 4a, 5a, 6a 7a, 17, 18, 19	The Pb and Cd were associated with mild increases in SRS scores at the 90th percentile of the SRS distribution. High gestational Pb concentration was weakly associated with the mild increases in SRS scores at the 90th percentile of the SRS distribution (*β*_*τ*=0.9_ = 0.99, 95% CI: −1.05, 3.02).
Skogheim et al. (2021)	Mother, Father and Child Cohort study (Norway) Parents = blood collection ∼17 weeks of gestation, Children = blood from umbilical cord at birth (*n* = 397 ASD cases, control = 1034)	As, Cd, Hg, and Pb Blood (ICP‐SFMS)	GM (μg/L): As: 1.65 Cd: 0.22 Hg: 1.17 Pb: 8.35	ICD‐10 codes F84.0, F84.1, F84.5, F84.8 or F84.9		1c, 2a, 3a, 7a, 8bcf, 9a, 10a, 11ei, 12a	The ASD was positively associated with the 2nd quartile of As [OR = 1.77 (CI: 1.26, 2.49)] in compared to 1st quartile. Highest quartiles of Cd showed higher risk of ASD [OR = 1.57 (CI: 1.07, 2.31)]. For Hg, all three quartiles (Q2, Q3, Q4) had significantly lowered risk of ASD compared to quartile 1.
Golding et al. (2018)	Population based/birth cohort study (UK) Mother‐child, Mother (4,484) Age: <18 weeks of gestation	Hg Whole blood (ICP‐DRC‐MS)	Median: 1.86 μg/L	ICD‐10, DAWBA	Children (3,885) 3‐11 years	1a, 6acf, 7a, 9a, 10a, 12	Total prenatal blood Hg levels was not associated with diagnosed autism (AOR 0.89; 95% CI 0.65, 1.22) per SD of Hg (*P* = 0.485). Maternal fish eating may modify the association with poor social cognition.
Ryu et al. (2017)	Birth cohort (Republic of Korea) Mother‐Child pairs, Gestational age: 12–20 weeks (early pregnancy), 28–42 weeks (late pregnancy), Children age: 2 and 3 years (458)	Total Hg Blood and cord blood (Gold amalgamation direct mercury analyzer)	Early pregnancy: GM: 3.53 μg/L Late pregnancy: GM: 3.30 μg/L μg/L Cord blood: GM: 5.52 μg/L 2 years of age: GM: 2.35 μg/L 3 years of age: GM: 2.16 μg/L	SRS	5 years	1a, 2a, 3a, 6a, 7a, 8bc, 9c, 11h	A 2‐fold increase of blood Hg levels at late pregnancy (*β* = 1.84, 95% CI: 0.39, 3.29, *p* = 0.0131), in cord blood (*β* = 2.24, 95% CI: 0.22, 4.27, *p* = 0.0303), and at 2 years of age (*β* = 2.12, 95% CI: 0.54, 3.70, *p* = 0.0087) and 3 years of age (*β* = 2.80, 95% CI: 0.89, 4.72, *p* = 0.0043) were positively associated with the SRS T‐scores. Only Hg levels at late pregnancy were associated with the SRS T‐scores (*β* = 1.71, *p* = 0.0357) and the ASD phenotype (relative risk = 1.34, *p* = 0.0073). After adjustment, only mercury levels at 3 years of age were associated with the SRS T‐scores (*β* = 3.27, p‐value = 0.0189); but not with the ASD phenotype.
Kim et al. (2016)	Cohort (Korea) Children, 7–8 years (2473)	Pb Blood (AAS)	GM at 7–8 years: 1.64 μg/dl, at 11–12 years: 1.58 μg/dl	ASSQ and SRS	11‐12 years	2, 3a, 6a, 7b, 8d, 9b, 11aj, 21f	Blood Pb concentrations at 7–8 years of age were positively associated with more autistic behaviors at 11–12 years of age according to the ASSQ (*β* = 0.151; 95% CI: 0.061, 0.242) and SRS (*β* = 2.489; 95% CI: 1.378, 3.600).
van Wijngaarden et al. (2013)	Cohort study (Seychelles) Mother‐child pairs (1784) Maternal hair samples collected at or near the time of birth. Maternal age: 15–45 years	MeHg Hair (Cold vapor atomic absorption)	Mean MeHg (ppm): 8.4	DSM‐IV and ICD‐10		1c, 2a	The null association was evident between prenatal MeHg exposure and total SCQ scores in the combined cohort in both linear and binomial regression models.
Cross‐sectional studies (*N* = 5)							
Dong et al. (2022)	Cross‐sectional (China) Children 2–13 years (ASD = 512)	Pb Blood (AAS)	Mean (μg/dl): Mild vs. Severe ASD: 2.58 vs. 3.25 ± 1.89	DSM‐5 CARS	2‐13 years		A high serum concentration of lead (*p* = 0.003) were statistically significantly associated with ASD severity
Dong et al. (2020)	Cross‐sectional (China) Children, 2–13 years (512)	Pb Blood (AAS)	Mean (μg/L): 39.09	ABC, CARS, ATEC		1, 4, 3ab, 21cg	A high Pb concentration in serum was significantly associated with ASD severity (OR = 1.038, 95% CI: 1.016–1.060). The Pb concentration was positively correlated with the total ABC score (*r* = 0.173, *p* = 0.004), the ABC sensory subscale score (*r* = 0.157, *p* = 0.008), the ABC self‐care subscale score (*r* = 0.161, *p* = 0.007), the ABC social interaction subscale score (*r* = 0.128, *p* = 0.032), the total CARS score (*r* = 0.202, *p* = 0.001) and the ATEC sociability subscale score (*r* = 0.139, *p* = 0.037).
Fiore et al. (2020)	Cross‐sectional (Italy) Children, 2–17 years (48)	As, Cd, Hg, and Pb Hair (ICP‐MS)	Median (μg/g): As: 0.082 Cd: 0.021 Hg: 0.338 Pb: 0.542	ADI‐R, ADOS			All the metals in hair (As, Cd, Pb levels) were positively correlated with the severity of ASD symptoms [As vs. ADOS‐P (rho: 0.31, *p* = 0.04), As vs. ADOS‐RR (rho: 0.31, *p* = 0.04); Cd vs. ADOS‐P (rho: 0.40, *p* = 0.01), Cd vs. ADOS‐RR (rho: 0.30, *p* = 0.05); Pb vs. ADOS‐P (rho: 0.47, *p* = 0.001), Pb vs. ADOS‐SA (rho: 0.31, *p* = 0.04). [ADOS‐P: Play and Creativity, ADOS‐SA: Social affect, ADOS‐RR: Restricted Repetitive Behavior]. A significant negative correlation was observed between hair Pb concentrations and cognitive level (full intelligence quotient) in ASD individuals (rho = −0.42; *p* = 0.003).
Gump et al. (2017)	Cross‐sectional (USA) Children, 9–11 years (203)	Hg (total) and Pb Blood (ICP‐MS)	Mean Pb (μg/dL): 0.98 Mean Hg (μg/L): 0.46	ASQ– Adolescent Version		1b, 2, 3a, 5, 6bd	The total ASQ score was not significantly associated with the increasing blood Hg and Pb levels before and after the adjustment with covariates (*p* > 0.10).
Geier et al. (2012)	Cross‐sectional (USA) Children, 1–6 years (18)	As, Cd, Hg, and Pb Hair (ICP‐MS)	Mean (μg/g): As: 0.074 Cd: 0.18 Hg: 0.33 Pb: 0.63	CARS			Increased hair Hg concentration significantly correlated with increasing ASD severity (rho = 0.58, *p* = 0.013, rho 95% CI: 0.15–0.82). No significant correlations were observed between any other of the hair toxic metals (As, Cd, Pb) examined and ASD severity.
Case‐control study (*N* = 25)							
Ouisselsat et al. (2024)	Case‐control (Morocco) Preschool children, 3–14 years (ASD: 102, control: 106)	Pb and Hg Tonail (ICP‐MS)	Pb: 3.345 vs. 2.871 μg/g Cd: 0.079 vs. 0.085 μg/g	DSM‐5 and CARS	3‐14 years	1, 2	Null association was found between Pb and Cd exposure levels and severity of ASD
Ouisselsat et al. (2023)	Case‐control (Morocco) Preschool children, 3–14 years (ASD: 107, control: 120)	Pb and Hg Hair (ICP‐MS)	Pb: 0.890 vs. 0.720 μg/g Hg: 0.193 vs. 0.200 μg/g	DSM‐5 and CARS	3‐14 years	1, 2	Null association was found between Pb and Hg exposure levels and severity of ASD
Saleh et al. (2023)	Case‐control (Egypt) 3–13 years (ASD = 32, TD = 30)	Cd and Pb Hair and Blood (ICP‐MS/MS)	ASD vs. TD (Median): Hair (mg/kg): Pb: 2.40 vs. 1.51 Cd: 2.91 vs. 0.47 Blood (mg/dl): Pb: 1.03 vs. 0.75 Cd: 0.77 vs. 0.31	DSM‐5 and CARS‐2‐HF	3‐13 years		Both blood and hair concentrations of Cd and Pb were significantly higher in ASD group in comparison to TD group. Blood Cd levels were significantly associated with CARS score (*p* < 0.05)
Abd Wahil et al. (2022)	Case‐control (Malaysia) Preschool children, 3–6 years (ASD: 81, Typically developed control: 74)	Pb Urine (ICP‐MS)	Pb: ASD vs. TD: 0.27 vs. 0.19 μg/dL	DSM‐5, ICD‐10	3‐6 years	1, 2	A Pb concentration was significantly lower in ASD than that of TD participants. Post interaction analysis showed that the odds of ASD reduced significantly by 0.1% with an increment of every 1.0 μg/dL urinary Pb, respectively).
Rezaei et al. (2022)	Case‐control (Iran) ASD vs. TD: 11.1 vs. 10.4 years (ASD = 44, TD = 35)	As, Cd and Pb Urine (ICP‐MS)	ASD vs. TD (μg/L): As: 17.93 vs. 12.18 Cd: 0.28 vs. 0.22 Pb: 6.19 vs. 2.11	DSM‐5	ASD vs. TD: 11.1 vs. 10.4 years	1, 2	The risk differences analysis suggest that As and Pb along with other trace elements remained significant risk factor for the ASD.
Gil‐Hernández et al. (2020)	Case‐control (Spain) Children, 2–6 years (Case: 54, Control: 54)	Hg Hair and Urine (AAS)	Mean in hair (μg/g): 8.26 Mean in urine (μg/L): 0.54	ADOS‐2, ADI‐R, PDDBI, CARS, Battelle Developmental test, and the SDQ.		1, 2, 8a, 21a	The null association were found between Hg concentrations in the ASD group as a whole (*p*: 0.739), or when they were subdivided into ASD‐AMR (*p*: 0.739) and ASD‐ANMR (*p*: 0.363)
Hawari et al. (2020)	Case‐control (Syria) Children, 3–12 years (ASD: 31, ADHD: 29, ASD‐C: 11, Control: 30)	Pb Blood (AAS)	Mean (μg/dl): 4.191	DSM‐5 criteria, CARS		1	A significant association was found between blood Pb levels and ASD group comparing with controls in the group of children aged 5 or less (OR = 2.475, 95% CI: 1.038–5.901, *p* = 0.041). The Pb levels were significantly higher in males compared with females in ASD group (*p* < 0.01).
Nabgha‐e‐Amen et al. (2020)	Retrospective case‐control (Pakistan) Children, 3–16 years (ASD: 90 Control: 76)	As, Cd, Hg and Pb Hair and urine (ICP‐MS)	Urinary As (mean: 36.67 vs. 15.65 μg/g cre), Urinary Hg (mean:1.13 vs. 0.11 μg/g cre), Urinary Pb (mean: 3.76 μg/g vs. 1.23 μg/g cre)	CARS, DSM‐V		8a, 21de	A significant positive association was found between hair As (*p* < 0.01, AOR = 18.29; 95% CI: 1.98, 169.05) and ASD. Urinary Hg (*p* < 0.05, AOR = 2.90; 95% CI: 1.39, 6.07), Pb (*p* < 0.05, AOR = 1.95; 95% CI: 1.01, 3.77) and As (*p* < 0.01, AOR = 1.04; 95% CI: 1.01, 1.06) were positively associated with the risk of ASD.
Rahbar et al. (2020)	Case‐control (Jamaica) Children, 2–8 years (ASD: 262, Control: 262)	As, Cd, Hg, and Pb Blood (ICP‐MS)	Mean (μg/L): As: 1.96, Cd: 0.20, Hg: 0.48, Pb: 2.21	ADOS, ADOS‐2, ADI‐R		For As: 1a, 3a, 6e, 11e For Cd: 3a, 11de, 12b For Hg: 1a, 3a, 11e For Pb: 1a, 3a, 6e, 11bg, 12b	ASD cases had lower GM blood concentrations of Pb (1.92 μg/dL vs. 2.34 μg/dL; *p* < 0.01) and median concentrations for Hg (0.64 μg/L vs. 0.81 μg/L; *p* = 0.01) in compared to typically developing controls. There was no significant differences were detected between ASD cases and typically developing controls with respect to median blood concentrations of As.
Ali et al. (2019)	Case‐control (Dhaka) Children, 3–16 years (Case: 25, Control: 25)	Pb Blood (XRF Spectrometry)	Mean Pb (μg/dl): 44.18 Median: 1.2	DSM‐IV TR			The mean blood Pb level in the ASD group was higher than the control group, though statistically not significant. A 48% of children belong to case group had blood Pb level ≥10 μg/dl (threshold level suggested by CDC, whereas in control group it was 24%.
Sehgal et al. (2019)	Case‐ control (India) Children, 3–12 years (ASD: 60, Control: 60)	As, Cd, Hg, and Pb Blood (ICP‐AES)	GM(μg/L): As: 0.3 Cd: 2.8 Hg: 6.2 Pb: 61.0	CARS 2nd edition			The null association was found between the tested heavy metals (As, Cd, Hg, Pb) and the severity of ASD [As: *r*_s_ = 0.3, *p* = 0.05; Cd: *r*_s_ = 0.2, *p* = 0.20; Hg: *r*_s_ = 0.2, *p* = 0.25; Pb: *r*_s_ = 0.1, *p* = 0.47]. The Pb was significantly higher in greater proportion of children with ASD [ASD vs. Control: 18.3 (11/60) vs. 1 (1.6), *p* = 0.002].
Rahbar et al. (2018)	Case‐control (Jamaica) Children, 2–8 years (ASD: 163, Control: 163)	As, Cd, Hg, and Pb Blood (ICP‐MS)	AM (μg/dl): As: 3.09, Cd: 0.23, Hg: 1.00, Pb: 2.97 GM (μg/dl): As: 2.48, Cd: 0.17, Hg: 0.66, Pb: 2.11	ADI‐R, ADOS			In comparison to typically developing controls, ASD cases had lower geometric mean blood concentrations of Pb (2.11 μg/dL vs. 2.68 μg/dL; *p* < 0.01) and Hg (0.66 μg/L vs. 0.78 μg/L; *p* < 0.05).
Adams et al. (2017)	Case‐control (USA) Children and adults, 2.5–60 years (Case: 67, Control:50)	As, Cd, Hg, and Pb Urine (ICP‐MS)	Urine (μg/g‐cre): As: 13.6 Cd: 0.34 Hg: 0.63 Pb: 0.59	ADOS, CARS‐2, PRO‐SAS, ABC, ATEC, PDD‐BI, SAS‐Parent, SSP, PGI‐R2			The total urinary metal was significantly associated with all 11 autism‐related assessments with cross‐validation R^2^ values ranging from 0.12 to 0.48.
Khaled et al. (2016)	Case‐control (Egypt) Children, 3–15 years, (Case: 40, Control: 60)	Hg and Pb Blood (AAS)	Hg (μg/L): 32.9 Pb (μg/L): 16.4	Diagnostic and Statistical Manual of Mental Disorders, DSM‐IV‐TR, ADI‐R, CARS.			The ASD patients showed significant higher levels of both Hg and Pb (*p* < 0.001) in the blood in comparison to the mean Hg and Pb concentrations of healthy controls and healthy siblings of the children with ASD. A significant positive correlation was observed between the levels of Hg in the blood and the CARS (*p* < 0.01).
Mostafa et al. (2016)	Case‐Control (Egypt) Children, 3–10 years (ASD: 84; Control: 84)	Hg Blood (AAS)	Blood Hg (μg/dl): 8 Median (μg/dl): 14	DSM‐IV CARS			The blood Hg levels were significantly higher in ASD children than in healthy control children (*p* < 0.001). Patients with severe ASD had significantly higher blood Hg (18.5 μg/dl) levels than patients with mild to moderate ASD (4.0 μg/dl, *p* < 0.001). A significant and positive linear relationship was observed between blood levels of Hg and values of CARS in ASD children (*r* ^2^ = 0.89; *p* < 0.0001).
McKean et al. (2015)	Population‐based case‐control (USA) Children, 2–5 years (Total: 257, ASD: 164, DD/AtD: 35, TD: 58)	MeHg Blood (ICP‐MS)	Mean 4.73 ppb	ADI‐R ADOS‐G module 1 or 2		1a, 2, 3a, 4b, 5b, 7d	The null association observed between natural log cumulative prenatal mercury exposure and the risk of autism (vs. Typical Development) after adjustment [OR = 1.03, 95% CI: 0.95, 1.12, *p* = 0.47].
Metwally et al. (2015)	Case‐control, cross‐sectional study (Egypt) Children, Mean age: 4.56 ± 2.1 y (Case: 55, Control: 75)	As, Cd, Hg, and Pb Urine (ICP‐MS)	Mean (μg/g cre): As: 175.6 Cd: 1.81 Hg: 11.03 Pb: 12.47	DSM‐V and CARS			The mean concentrations of heavy metals (As, Cd, Hg, Pb) were significantly higher in ASD subjects than in controls (For Cd, *p* < 0.05 and for As, Hg, Pb, *p* < 0.01). A positive but non‐significant correlation was found between the measured heavy metals (As, Cd, Hg, Pb) and severity of autism which was assessed by CARS score [As vs. CARS: *r* = 0.098; Cd vs. CARS: *r* = 0.122; Hg vs. CARS: *r* = 0.056; Pb vs. CARS: *r* = 0.141].
Hodgson et al. (2014)	Case‐control (Oman) Children, Mean age: 5.3 years (Case: 27, Control: 27)	Hg Hair (ICPMS)	Mean (μg/g): 6.93	CARS			A 10‐fold elevated level of Hg was significantly associated with ASD cases (6.93 ± 0.36 μg/g, *p* < 0.0001) than in control groups (0.611 ± 0.033 μg/g).
Rahbar et al. (2014)	Case‐control (Jamaica) Children, 2–8 years (ASD: 100, Control: 100)	Pb Blood (ICP‐MS)	GM (μg/dl): 2.25	DSM‐IV‐TR, CARS		1a, 3a, 6e, 11bg, 12b	A significant association was observed between blood Pb concentrations and ASD status, with lower blood Pb concentrations in ASD cases (2.25 μg/dL for ASD vs. 2.73 μg/dL for TD; *p* < 0.05) through univariable analysis before adjustment. After adjustment for potential confounders, there were no significant differences between adjusted geometric mean blood Pb concentrations of ASD cases and controls (2.55 μg/dL vs. 2.72 μg/dL, *p* = 0.64).
Yau et al. (2014)	Case‐control (USA) Mother‐baby pairs, Maternal age (mean): ASD: 30.7 years, DD: 28.5 years, GP (General population): 28.3 (ASD = 84, Developmental delay (DD) = 49, Control = 159)	Total Hg blood (ICP‐MS)	GM (ng/ml): Maternal serum: 0.48 Newborn Blood spots: 3.52 AM (ng/ml): Maternal serum: 0.85 Newborn Blood spots: 6.49	DSM‐IV	Within 24–48 hr of birth	1ab, 2a, 4b, 5a, 7bc, 8e, 12a	Maternal serum and neonatal blood Hg levels were positively as well as significantly correlated with ASD severity [*r* = 0.3928, *p* < 0.001]. The null associations were observed between ASD and Hg levels in maternal serum samples (ASD vs. GP: OR [95% CI] = 0.96 [0.49–1.90]; ASD vs. DD: OR [95% CI] = 2.56 [0.89–7.39]) and newborn blood samples (ASD vs. GP: OR [95% CI] = 1.18 [0.71–1.95]; ASD vs. DD: OR [95% CI] = 1.96 [0.75–5.14]).
Adams et al. (2013)	Case‐control (Arizona‐USA) Children, 5–16 years (Case: 55, Control: 44)	As, Cd, Hg, and Pb Blood and urine (ICP‐MS)	Whole blood (μg/L): As: 3.30, Cd: 0.64, Hg: 0.75, Pb: 10.4 RBC (ng/g): As: 4.3, Hg: 1.2, Pb: 19 Urine (μg/g‐cre): As: 30.8, Cd: 0.282, Hg: 2.58, Pb: 0.57	PDD‐BI, ATEC, SAS		1, 2, 11f, 13, 14, 15, 16, 20	High concentrations of blood Hg (both WB and RBC) were significantly associated with two out of three autism severity scales [WB‐ATEC: 0.36 (0.008), WB‐SAS: 0.34 (0.01)]; [RBC‐ATEC: 0.38 (0.005), RBC‐SAS: 0.31 (0.03)]. A strong association of toxic metals levels (As, Cd, Pb, Hg) with variation in the degree of severity of autism was found for all the autism severity scales (adjusted R^2^ of 0.38–0.47, *p* < 0.0003).
Blaurock‐Busch et al. (2012)	Case‐control (Saudi Arabia) Children, 3–9 years (Case: 44, Control: 146)	As, Cd, Hg, and Pb Hair (ICP‐MS)	Mean (mg/kg): As: 2.94 Cd: 0.62 Hg: 3.35 Pb: 4.56	CARS			A high concentration of Pb in hair was positively associated with verbal communication (*p* = 0.020) and general impression (*p* = 0.008) A high Hg concentration was associated with impaired object use (*p* = 0.040) and auditory response (*p* = 0.021)
De Palma et al. (2012)	Cross‐sectional case‐control study (Italy) Children, Mean age: 9.00 ± 4.05 years (ASD: 44, Controls: 61)	Pb, Hg, As, and Cd Hair (ICP‐MS)	Median (μg/g): As: 0.03 Cd: 0.01 Hg: 0.50 Pb: 1.30	DSM‐IV, CARS		1, 2	The null association was found between hair metals and autism.
Lakshmi Priya and Geetha (2011)	Case‐control (India) Children, 4–12 years (Case: 45, Control: 50)	Hg and Pb Hair and nail (Cold vapor method using MHS‐15, AAS)	In Hair: Mean Pb (μg/g): LFA: 17.97; MFA: 3.24; HFA: 2.04 Mean Hg (μg/g): LFA:3.09; MFA:1.10; HFA: 0.65 In Nail: Mean Pb (μg/g): LFA: 26.38; MFA: 17.68; HFA: 16.48 Mean Hg (μg/g): LFA:5.12; MFA: 4.37; HFA: 2.57	Check of Autism in Toddlers based on CARS score			Significant elevated levels of both Pb and Hg in the hair and nail samples were observed in LFA group when compared with HFA group. The Pb and Hg concentrations in the hair and nail were significantly correlated with the degree of severity of autism [Pb (Hair) vs. CARS: *r*_s_ = 0.820, *p* < 0.001; Pb (Nail) vs. CARS: *r*_s_ = 0.827, *p* < 0.001; Hg (Hair) vs. CARS: *r*_s_ = 0.748, *p* < 0.001; Hg (Nail) vs. CARS: *r*_s_ = 0.764, *p* < 0.001].
Geier et al. (2010)	Case‐control (USA) Children, 2.3–15.2 years (Case: 83, Control: 89)	Hg (total) Blood (Cold vapor Hg analysis system)	Mean (μg/L): 21.4	ICD‐9 ASD		1, 2, 21b	High Hg levels (>15 μg/L) were significantly (*p* < 0.0005) associated with the risk of a subject being diagnosed with an ASD in comparison to controls (OR = 6.4) after adjustment.

y, year; cre, creatinine; AAS, atomic absorption spectroscopy; MeHg; methyl mercury; ABC, autism behavior checklist; ADI‐R, Autism diagnostic interview‐revised; ADOS, autism diagnostic observation schedule; GM, geometric mean; AM, arithmetic mean; ASSQ, autism spectrum screening questionnaire; ATEC, autism treatment evaluation checklist; BMI, body mass index; CARS, childhood autism rating scale; CGI‐S, clinical global impressions scale; DAWBA, development and well‐being assessment; DSM‐5, diagnostic and statistical manual of mental disorders, Fifth edition; GC‐MS, Gas chromatography‐mass spectrometry; GM, geometric mean; HFA, high‐functioning autism; ICD, international classification of diseases; ICP‐AES, inductively coupled plasma atomic emission spectroscopy; ICP‐MS, Inductively coupled plasma mass spectrometry; ICP‐SFMS, inductively coupled plasma sector field mass spectrometry; LFA, low functioning autism; MFA, medium functioning autism; MHS, mercury hydride System; PDD‐BI, pervasive developmental disorder behavior inventory; SAS, severity of autism scale; SDQ, Strengths and Difficulties Questionnaire; SRS, social responsiveness scale; SSP, safe and sound protocol; XRF, X‐ray fluorescent. 1 Age (^a^maternal/paternal age, ^b^child age, ^c^maternal/paternal age at delivery); 2 Gender/sex (^a^child sex); 3 Education level (^a^maternal/paternal education, ^b^caregivers education); 4 Residence (^a^maternal city of residence, ^b^maternal birthplace (USA/Mexico/other)); 5 Race/ethnicity (^a^maternal ethnicity, ^b^child's race/ethnicity); 6 Socioeconomic status (^a^household income/housing tenure, ^b^annual household income, ^c^household crowding, ^d^occupation, ^e^car ownership by the family, ^f^family adversity index); 7 Information of pregnancy (^a^parity, ^b^gestational age at delivery, ^c^gestational age at maternal blood draw, ^d^payment method for child delivery); 8 Physique (^a^BMI, ^b^pre‐pregnancy body mass index, ^c^birth weight, ^d^low birth weight, ^e^mother's body weight at blood draw, ^f^small for gestational age); 9 Smoking (^a^smoking in pregnancy, ^b^fetal and environmental tobacco smoke exposure, ^c^maternal tobacco smoking); 10 Alcohol consumption (^a^prenatal alcohol consumption); 11 Food (^a^breast feeding, ^b^consumption of shellfish‐lobsters, crabs, ^c^source of drinking water, ^d^consumption of yam, sweet potato, or dasheen/carrot or pumpkin/callaloo, broccoli, or pak choi/cabbage/fried plantain/boiled dumpling/white bread, ^e^the frequency of seafood consumption per week, ^f^special diets, ^g^teflon (pots, pans, and dishes used for cooking), ^h^maternal fish intake during pregnancy, ^i^dietary iodine intake (μg/day), ^j^fish intake of the children at the time of Pb level measurement); 12 Life events (^a^child's birth year, ^b^parish of child's birth (in “Kingston, St. Andrew, or St. Catherine parishes vs. other parishes”)); 13 Clinical variables (speech delay, cognitive, deficit, infantile psychosis, and hyperkinetic syndrome); 14 Diagnosis; 15 Medications; 16 Nutritional supplements; 17 Folic acid supplementation; 18 Caregiver environment score; 19 Relationship status; 20 ASD rating scale (^a^PDD‐BI, ^b^ATEC, ^c^SAS); 21 Others (^a^urine creatinine level, ^b^year of sample collection, ^c^serum concentrations of vitamin D, copper, lead, ^d^trace elements concentrations, ^e^glutathione S‐ transferase GSTT1 and GSTM1 presence/absence; ^f^log‐transformed blood mercury concentration, ^g^gastrointestinalproblems).

## 3 Results

A total of 36 human epidemiological studies explored the associations of As, Cd, Hg, and Pb with ASD‐related outcomes. Most of the studies belong to the case‐control group, and the rest were cohort or cross‐sectional studies (Table 1). An analytical synthesis of the epidemiological evidence examining associations between heavy metals and ASD‐related outcomes across studies is shown in Table 2. It summarizes the direction of associations for each heavy metal with ASD, and the number and proportion of study types contributing to positive findings. Table 3 represents a summary of the reported associations between heavy metals and ASD according to exposure metrics. Pb and Hg were the most frequently investigated metals and showed the greatest variability in findings, with positive, null, and inverse associations reported across different biological matrices. Cd and As were examined less frequently, and the findings were mostly inconsistent. In the majority of studies, blood samples were collected to determine toxic metal concentration along with urine, hair, and nail samples, while fewer studies utilized cord blood, maternal blood, neonatal blood spots, toenails, or nails. Overall, substantial heterogeneity was observed in the direction of associations across metals and types of exposure metrics. The ICP‐MS and AAS were mostly applied to measure the concentrations of heavy metals in blood (whole, serum, or plasma), urine, hair, and nails. Mostly used tools to assess ASD and its severity among children, and adolescents include autism treatment evaluation checklist, severity of autism scale (SAS) (Adams et al., 2013), autism diagnostic observation schedule (Fiore et al., 2020), childhood autism rating scale (CARS) (Blaurock‐Busch et al., 2012; Dong et al., 2022), social responsiveness scale (SRS) (Kim et al., 2016), autism behavior checklist (Dong et al., 2022), autism diagnostic interview‐revised (Fiore et al., 2020), pervasive developmental disorders behavior inventory (Adams et al., 2013), autism spectrum quotient (Gump et al., 2017), diagnostic and statistical manual of mental disorders, fourth edition, text revision (DSM‐IV‐TR) (Khaled et al., 2016). According to CARS, an individual is non‐autistic if the total score remains 15–29.5, whereas mild to moderate as well as moderate to severe autism are considered when the total scores fall in the range of 30–36.5 and 37–60, respectively (Blaurock‐Busch et al., 2012; Geier et al., 2012). Similarly, SRS is also a 65‐item behavioral rating scale that gives scores ranging from 0 to 195 to evaluate the severity of autism symptoms (van Wijngaarden et al., 2013).

**Table 2:** Direction of Associations Between Heavy Metal Exposure and ASD‐Related Outcomes, and Proportions of Study Found Positive Relationships

Heavy metal	Total studies (n)	Positive association *n* (%)	Null association *n* (%)	Inverse association *n* (%)	Positive findings by study design *n* (%)
Pb	27	16 (59.3)	8 (29.6)	3 (11.1)	Case‐control: 9 (56.3) Cross‐sectional: 4 (25.0) Cohort: 3 (18.7)
Hg	26	12 (46.2)	11 (42.3)	3 (11.5)	Case‐control: 7 (58.3) Cross‐sectional: 2 (16.7) Cohort: 3 (25.0)
Cd	15	7 (46.7)	8 (53.3)	–	Case‐control: 5 (71.4) Cross‐sectional: 1 (14.3) Cohort: 1 (14.3)
As	14	7 (50.0)	7 (50.0)	–	Case‐control: 5 (71.4) Cross‐sectional: 1 (14.3) Cohort: 1 (14.3)

**Table 3:** Summary of Reported Associations Between Heavy Metal and ASD According to the Types of Exposure Metrics

Heavy metal	Major exposure matrices	Total counted studies (n)	Positive association *n* (%)	Null association n (%)	Inverse association *n* (%)
Pb	Blood	12	5 (41.7)	5 (41.7)	2 (16.7)
	Hair	3	1 (33.3)	2 (66.7)	–
	Nail	1	–	1 (100.0)	–
	Urine	5	4 (80.0)	0 (0.0)	1 (20.0)
Hg	Blood	12	4 (33.3)	5 (41.7)	3 (25.0)
	Cord Blood	2	–	2 (100.0)	–
	Hair	5	1 (20.0)	4 (80.0)	–
	Urine	4	2 (50.0)	2 (50.0)	–
Cd	Blood	4	2 (50.0)	2 (50.0)	–
	Hair	2	1 (50.0)	1 (50.0)	–
	Nail	1	–	1 (100.0)	–
	Urine	3	1 (33.3)	2 (66.7)	–
As	Blood	3	1 (33.3)	2 (66.7)	–
	Hair	2	1 (50.0)	1 (50.0)	–
	Urine	4	3 (75.0)	1 (25.0)	–

### 3.1 Findings From Cohort Studies

Among six cohort studies, the mother‐child pair ranged from 458 to 1784 and was enrolled in five studies (Table 1). The remaining study recruited a large number of mothers and children: 4,484 and 3,885, respectively (Golding et al., 2018). The mother's age ranges from ≥18 years to ≥42 years. All four heavy metals were measured in maternal biospecimens during gestational periods (ranging from 6 to 42 weeks) in two studies (Alampi et al., 2021; Skogheim et al., 2021). Blood and cord blood Hg levels were determined in two birth cohorts (Golding et al., 2018; Ryu et al., 2017), and methyl Hg was determined in the hair specimens in one study (van Wijngaarden et al., 2013). The geometric mean concentrations of As, Cd, Hg, and Pb ranged from 0.82 to 1.65 μg/L, 0.20–0.22 μg/L, 1.17–3.53 μg/L, and 1.64–8.35 μg/L, respectively. One study showed that the blood arsenic level of pregnant participants was positively associated with ASD symptoms in children (Skogheim et al., 2021). Maternal blood Hg or hair methyl Hg levels showed null and negative associations with ASD severity and risk in three studies (Golding et al., 2018; Skogheim et al., 2021; van Wijngaarden et al., 2013), whereas a significant positive association was observed between blood Hg concentrations measured at late pregnancy as well as 2‐ and 3‐year‐old children and SRS T‐scores, and ASD phenotypes at 5‐year‐old children's (Ryu et al., 2017). The blood Cd and Pb levels were positively associated with a mild increase in SRS scores and ASD risk (Alampi et al., 2021; Skogheim et al., 2021). In another cohort study involving only children between the ages of 7 and 8 years, blood Pb concentrations were positively associated with more autistic behaviors at 11–12 years of age according to the autism spectrum screening questionnaire and SRS scales (Kim et al., 2016).

### 3.2 Findings From Cross‐Sectional Studies

A total of five cross‐sectional epidemiological studies investigated the associations of heavy metals (As, Cd, Hg, Pb) with ASD symptoms or severity in 1–17 years (Table 1). The participant numbers ranged from 18 to 512. Blood and hair Hg and Pb concentrations were significantly associated with ASDs severity (Dong et al., 2020, 2022; Fiore et al., 2020; Geier et al., 2012; Gump et al., 2017), whereas the other two metals showed inconsistent association (positive, negative, and null) with ASDs and ASD severity in children after adjusting for potential covariates (Dong et al., 2020; Fiore et al., 2020; Geier et al., 2012; Gump et al., 2017).

### 3.3 Findings From Case‐Control Studies

A total of 25 out of 36 studies were case‐control in study design. Almost all these studies enrolled two to 16 years old. Among them, in six studies, the As, Cd, Hg, and Pb levels were measured in the blood and/or urine of the study participants, and in hair samples in four studies. The rest of the studies measured either Pb or Hg concentrations in blood, urine, and hair samples of the study participants. A total of 18 studies measured either total or free Hg in the blood or hair. Only one study measured Hg and Pb in nail samples of the study participants (Lakshmi Priya & Geetha, 2011). Blood and urine Hg concentrations were reported to be positively associated with ASD severity and risk in both children and adolescents assessed by several ASD assessment tools (Adams et al., 2013; Adams et al., 2017; Geier et al., 2010; Khaled et al., 2016; Metwally et al., 2015; Mostafa et al., 2016; Nabgha‐e‐Amen et al., 2020; Yau et al., 2014). A few studies reported null associations of urinary or blood Hg concentrations with ASD severity (Gil‐Hernández et al., 2020; McKean et al., 2015; Sehgal et al., 2019) whereas the associations were positive or null between hair and nail Hg concentrations and ASDs in children’s (Blaurock‐Busch et al., 2012; De Palma et al., 2012; Geier et al., 2012; Gil‐Hernández et al., 2020; Hodgson et al., 2014; Lakshmi Priya & Geetha, 2011; Nabgha‐e‐Amen et al., 2020; Ouisselsat et al., 2023; Ouisselsat et al., 2024). Only two studies reported negative associations between the blood Hg concentrations and ASD severity in 2–8 years children's (Rahbar et al., 2018, 2020). A total of 17 studies quantified Pb in blood, urine, or hair samples of children. The majority of them reported significant positive associations of blood and hair Pb concentrations with ASD severity measured in different assessment scales, and ASD risk (J. Adams et al., 2017; J. B. Adams et al., 2013; Blaurock‐Busch et al., 2012; Hawari et al., 2020; Khaled et al., 2016; Lakshmi Priya & Geetha, 2011; Metwally et al., 2015; Rezaei et al., 2022). A few studies reported null associations between blood or hair Pb concentrations and ASD severity in children (De Palma et al., 2012; Geier et al., 2012; Sehgal et al., 2019), whereas others reported negative associations (Abd Wahil et al., 2022; M. H. Rahbar et al., 2018, 2020). A total of 10 case‐control studies determined arsenic in blood, urine, and hair samples of the study children. Among them, 4 reported positive associations (J. Adams et al., 2017; J. B. Adams et al., 2013; Metwally et al., 2015; Nabgha‐e‐Amen et al., 2020), and the remaining studies found null associations of blood, urine, and hair As concentrations with ASD scores in children (Blaurock‐Busch et al., 2012; De Palma et al., 2012; Geier et al., 2012; Rahbar et al., 2018, 2020; Sehgal et al., 2019). A total of 10 case‐control studies investigated the associations of blood and urine Cd concentrations with several ASD assessment scales in children. The majority of the studies found null associations between blood or urine Cd concentrations and ASDs (Blaurock‐Busch et al., 2012; De Palma et al., 2012; Geier et al., 2012; Nabgha‐e‐Amen et al., 2020; Rahbar et al., 2018, 2020; Sehgal et al., 2019), whereas only three studies reported positive associations between blood or urine Cd levels and ASDs (J. Adams et al., 2017; J. B. Adams et al., 2013; Metwally et al., 2015).

Overall, no studies reported the age‐dependent associations of heavy metals and ASDs. Sex stratified reports were also distracted. All of the studies investigated the associations of single metal exposure with ASDs at a time. There was no study that explored the mixture effects of the metals on ASDs severity or risk. A few studies, ethnicity/race were adjusted as covariates, but none of the studies investigated the ethnicity‐stratified associations. Almost half of the studies did not adjust for the candidate confounders, whereas others adjusted for non‐specific covariates without considering the causal path.

## 4 Discrepancies, Research Gaps, and Future Perspectives

In the present review, we summarized a total of 36 existing original research works and explored their findings irrespective of age, sex, and geographic regions. We found inconsistent associations between prenatal and early to late childhood blood, urine, and hair As and Cd exposure levels and ASDs. Prenatal and early childhood acute and chronic Pb and Hg exposure are associated with ASDs in children. The age, sex, and ethnicity‐stratified associations of heavy metals with ASDs were not evident.

We found a limited number of prospective cohort studies; the sample size was inadequate for several studies. The small sample size may exhibit statistical changes or bias in the data examined, and some of the observed results, too (Geier et al., 2012). The samples were collected from specific geographic regions on specific populations for each study which might be different in other places. Thus, the selection bias might present and the results might not be generalized (Adams et al., 2013). Most of the samples were collected from a single time point which did not involve early exposure data. Only two study diagnosed the ASD using ICD‐9 and ICD‐10 disease code (Geier et al., 2012; van Wijngaarden et al., 2013), whereas all others assessed ASDs by several ASD assessment scales; varies among studies and age of study participants. Most of the cases cross‐verification was absent in‐patient ASDs diagnosis. Assessment of ASDs in children with one or two scales might be overestimated due to the lack of standardization (Alampi et al., 2021). For example, the neurodevelopmental screening tools (the TONI and Beery VMI) were not standardized and may have examiner bias (Al‐Saleh et al., 2020). Thus, biasness might be present during complete case analysis which might be great drawbacks to define the causal relationships.

The exposure metrics or biospecimens (blood, urine, hair, and nail) were varied among the existing studies (Table 1). Most of the studies assessed the heavy metal exposure levels in blood and urine. Typically, blood and urine concentrations of heavy metals might indicate current exposure, ideally through drinking water and food items they consumed (Lindstedt et al., 1979; Zhang et al., 2015). In contrast, hair and nail exposure indicate long‐term or chronic exposure (Lakshmi Priya & Geetha, 2011). Depending on geographic regions (endemic and non‐endemic) and contaminated food habits, the exposure levels of blood and urinary heavy metals can be largely altered (Adams et al., 2013; Metwally et al., 2015). The associations between acute heavy metals exposure and ASDs might have chance of some potential recall bias than that of the association between chronic exposure and ASDs (Rahbar et al., 2018). Most of the present study considers only one exposure specimen, mostly either blood/urine or hair. Only a few studies consider both acute exposure (measured in blood or urine) and chronic exposure (measured in hair or nails), and assess the relationships between heavy metals and ASDs, which might demonstrate strong evidence (Lakshmi Priya & Geetha, 2011; Nabgha‐e‐Amen et al., 2020).

The exposure levels of heavy metals among urine, blood, and hair samples of the study participants among the 36 studies, irrespective of age, were comparable to or relatively lower than that of the reference limits or guidance values recommended by different regulatory organizations (Saravanabhavan et al., 2017; Yadav et al., 2019). Only a limited number of studies reported the higher concentrations of heavy metals in blood, urine, and hair samples of children (Adams et al., 2013; Blaurock‐Busch et al., 2012; Geier et al., 2010; Khaled et al., 2016; Metwally et al., 2015; Yau et al., 2014). We found two studies, both determined blood Pb concentrations, but one study found positive associations that had high Pb concentrations (Dong et al., 2020), and the other study found negative associations that had low Pb concentrations (Gump et al., 2017), although the study participants and ASD assessment scales were different. From the toxicological viewpoint, the association between exposure and outcome largely depends on doses and duration of exposure. Higher levels of toxic metals in blood or urine suggest a combination of increased exposure, increased absorption, and/or decreased fecal excretion (Balali‐Mood et al., 2021; Jaishankar et al., 2014). In contrast, a few studies found similar associations between prenatal or early childhood heavy metal concentrations and ASD irrespective of participant exposure levels, exposure time, age, and geographic regions (Fiore et al., 2020; D. A. Geier et al., 2012; Rahbar et al., 2018; Skogheim et al., 2021). The inconsistent associations among the studies might be explained by methodological variations, particularly the characteristics of the study populations. Other potential reasons might include the timing and duration of metal exposure, although all of these are persistent in the environment, and racial or geographic differences among the participants. There is clear evidence of sex and age‐specific ethnic and racial differences of ASDs in children (Pham et al., 2022; Yuan et al., 2021). However, none of the existing studies evaluated the ethnicity‐stratified associations between metal exposure levels and ASD, although the reasons for racial and ethnic differences to induce ASD are largely unknown (CDC, 2018). Possible reasons might be the different patterns of metabolic activity, endocrine, and genetic susceptibility among racial and ethnic groups (Cheng et al., 2017; De Luca, 2020; Rylaarsdam & Guemez‐Gamboa, 2019). Only a few studies adjusted the race/ethnicity as cofounder to assess the relationships between heavy metals and ASD, which may definitely strengthen the findings (Alampi et al., 2021; Gump et al., 2017; McKean et al., 2015; Yau et al., 2014). In contrast, a significant number of studies did not consider any confounders to adjust the findings. None of the studies define the selection of confounders used for model adjustment/or did not consider the directed acyclic graph to select the appropriate confounder, and remove the colliders and mediators as stated elsewhere (Tennant et al., 2021).

All the investigated associations were generated by normal linear regression analyses, which make the results straightforward and easily interpretable. The emerging concern is that generalized linear regression can provide a simple relationship between a single exposure and outcome at a time, but cannot explore the joint/combined effect of mixture exposure (Zhang et al., 2019). In addition, to study causality, researchers need to consider mixed environmental exposures and their complex nonlinear interactions (Mohanto et al., 2021). Ignoring the joint effects of other metals could contribute to false‐positive or false‐negative results (Czarnota et al., 2015). Moreover, mediation or path analysis to evaluate the mediator's role of biochemical, genetic, or neurological factors on the association of single metal exposure and ASD can strengthen the findings in the future and is essential to establish the mechanistic pathway. We did not find any study that investigated the associations between cumulative exposure to metals and ASDs using a joint/aggregated effect assessment statistical approach. The application of multipollutant statistical models like quantile‐g‐computation (qgcomp), generalized weighted quantile sum regression (gWQS), and Bayesian kernel machine regression (BKMR) to clarify the joint effects of mixed metals could be well accepted and utilized to explore the relationships of a cumulative metal exposure with ASD. Additionally, assessment of both chronic exposure to single and mixed heavy metals and ASD severity trajectory might shed deeper light on causal relationships (Nishimura et al., 2019).

Most of the studies did not identify the exposure sources, which is very important since some heavy metals come from rice, vegetables and juices, processed fruit products, and so on (Fathabad et al., 2018; Hasan et al., 2022; Mawari et al., 2022). Although the exposure sources of metals were already reported in several existing studies, the study‐specific sources might be suitable to explain the inconsistent associations among the studies of different geographic regions, since diets and nutrition are also reported to be associated with ASDs (Doreswamy et al., 2020).

Last but not least, the large prospective cohort study with a significant number of study participants may be designed to explore the association of both single and combined exposure to heavy metals and ASDs in the future. To design a new study, researchers should minimize the selection bias and exposure assessment bias through biomonitoring of appropriate biospecimens to clarify the current exposure and/or chronic exposure. Diagnosis of ASD should be conducted following well‐accepted and validated assessment tools, possibly through clinical diagnosis. Besides these, researchers should consider demographic and lifestyle factors along with diets that could have potential confounding effects to strengthen the findings and draw causal relationships.

### 4.1 High Priority Research Gaps

Several high‐priority research gaps emerge from the current literature. First, there is a notable scarcity of prospective cohort studies that incorporate repeated measures of exposure. Most available evidence comes from cross‐sectional or case‐control designs that rely on single‐time‐point exposure matrices. To establish temporal relationships and support causal inference, large, longitudinal birth cohorts with repeated biomonitoring of heavy metals across critical developmental windows—namely the prenatal period, infancy, and early childhood—are needed. Second, no study to date has examined the joint or combined effects of arsenic, cadmium, mercury, and lead on ASD risk or severity using advanced multipollutant statistical models such as BKMR, gWQS regression, or qgcomp, because human populations are exposed to complex mixtures of metals rather than to single agents. Third, formal evaluations of effect modification by age, sex, or race/ethnicity remain absent from the existing literature. Given the well‐documented male predominance in ASD prevalence (approximately 4:1) and the recognized racial and ethnic disparities in ASD diagnosis, addressing this gap should be a high priority for future research. Fourth, although toxicological studies have proposed plausible mechanistic pathways—including oxidative stress and neuroinflammation—very few epidemiological investigations have measured relevant biomarkers (e.g., F2‐isoprostanes for oxidative stress, cytokines for neuroinflammation) that would allow mediation or path analysis. Integrating such mechanistic biomarkers into epidemiological study designs is essential for establishing causal pathways. Finally, the specific sources of heavy metal exposure and potentially modifiable risk factors remain largely unidentified. Most studies do not ascertain whether exposure originates from diet, drinking water, air pollution, or other sources, nor do they systematically evaluate effect modifiers such as nutritional status or genetic polymorphisms in detoxification enzymes. Identifying modifiable factors is a key step toward developing effective prevention strategies.

## 5 Conclusions

The association between heavy metal exposure and ASD appears to be both inconsistent and metal‐specific. For arsenic and cadmium, the evidence is null: nearly 50% of studies on arsenic and 53% on cadmium reported no significant associations. In contrast, mercury showed considerable heterogeneity—about 46% of studies reported positive associations, 42% found null results, and 12% observed inverse associations. Lead displayed a somewhat more consistent positive signal, with roughly 60% of studies reporting positive associations, 30% null findings, and 10% negative ones. Taken together, our review does not support a blanket conclusion that heavy metals as a class are associated with ASD. Rather, the evidence suggests that lead and mercury may increase ASD risk or symptom severity in certain populations and under specific exposure conditions. However, these findings must be interpreted with caution, given the marked heterogeneity across studies, the potential for residual confounding, and the methodological limitations that characterize much of the existing literature.

## Conflict of Interest

The authors declare no conflicts of interest relevant to this study.

## Data Availability

We did not generate any new data. Therefore, there are no data to share.
